# Navigating the Diagnostic Maze: A Case Report of Immunoglobulin G4-Related Disease

**DOI:** 10.7759/cureus.64502

**Published:** 2024-07-14

**Authors:** Muhammad Ayub Khan, Naeem Ullah, Salman Khan, Wajeeh Ur Rehman, Muzammil Ahmad Shah

**Affiliations:** 1 Internal Medicine, Saidu Group of Teaching Hospitals, Swat, PAK; 2 Rheumatology, Saidu Group of Teaching Hospitals, Swat, PAK

**Keywords:** immunoglobulin g4, immunoglobulin g4-related disease, igg-4 related disease, glucocorticoid, uncertain pathology, glucocorticoid therapy, diagnostic challenges, systemic fibroinflammatory condition

## Abstract

Immunoglobulin G4-related disease (IgG4-RD) is a systemic fibroinflammatory condition characterized by significant infiltration of immunoglobulin G4 (IgG4)-positive plasma cells within affected tissues, with or without elevated serum IgG4 levels. The prevalence of IgG4-RD remains largely undetermined due to diagnostic challenges, as the condition is frequently unrecognized or misdiagnosed. This report describes a case of a 63-year-old man who was ultimately diagnosed with this rare condition after an extensive two-year period of elusive symptoms. Initially presenting with intermittent body pains and fluctuating fever, his condition progressively evolved to include severe right orbital swelling with marked tenderness and ecchymosis, recurrent non-tender nodules on his arm, and diminished vision. A detailed review of his medical history prompted the consideration of IgG4-RD, leading to the measurement of serum human IgG4 levels, which were found to be significantly elevated at 1504 mg/L (normal range: 39.2-864 mg/L). Following his diagnosis, treatment with glucocorticoids (0.6 mg/kg for one month) was initiated, resulting in a positive clinical response. This case emphasizes the critical importance of considering less common conditions in the differential diagnosis of patients presenting with complex, multi-system symptoms.

## Introduction

Immunoglobulin G4-related disease (IgG4-RD) is a systemic, fibroinflammatory condition characterized by significant infiltration of immunoglobulin G4 (IgG4)-positive plasma cells within various tissues, possibly accompanied by elevated plasma levels of IgG4 [1]. This pathological process often leads to storiform fibrosis and tumefactive lesions, causing chronic damage that can potentially affect any organ system [2]. Over time, multiple syndromes previously considered distinct clinical entities have been reclassified as manifestations of IgG4-RD. These include Mikulicz disease [3], chronic sclerosing sialadenitis (Kuttner's tumor) [4], Riedel’s thyroiditis, mediastinal fibrosis, retroperitoneal fibrosis (Ormond’s disease), periaortitis, idiopathic hypocomplementemic tubulointerstitial nephritis, multifocal fibrosclerosis, and inflammatory pseudotumors [5,6]. The broad spectrum of involvement underscores the disease's systemic nature and its capacity for diverse organ-specific presentations.

The primary objective of this case report is to delineate the diagnostic challenges and therapeutic considerations of IgG4-RD through the detailed presentation of a complex case involving a middle-aged male. The patient exhibited various symptoms, including fever, weight loss, a swollen left leg, non-tender reddish nodules on both arms, and significant right orbital swelling. This case highlights the critical need for heightened clinical awareness and a systematic diagnostic approach to effectively identify and manage IgG4-RD, given its potential to mimic various other pathologies and its profound impact on patient outcomes.

## Case presentation

A 63-year-old male with no known comorbidities presented with a perplexing array of symptoms, including waxing and waning non-tender skin nodules, intermittent body pains, and sporadic fever, first noted 18 months prior. Remarkably, his symptoms were not accompanied by weight loss. Despite receiving symptomatic treatment for four months, no definitive diagnosis was reached, and his condition showed no improvement. Consequently, he was admitted to a tertiary healthcare facility for an in-depth evaluation to ascertain the cause of his symptoms.

**Figure 1:**
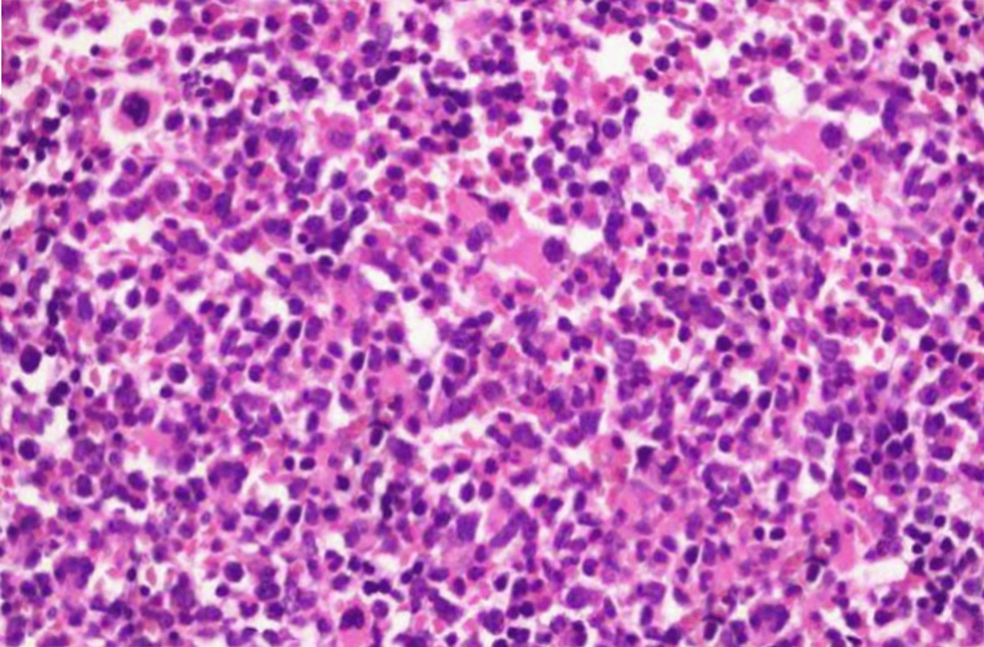
The image showing hypercellular bone marrow and the absence of abnormal cell clusters or blast cells.

Upon admission, both his general and systemic physical examinations were unremarkable. Further diagnostic investigations included bone marrow and trephine biopsy, demonstrating adequate hypercellularity (40-50%) with a small proportion of ringed sideroblasts and plasma cells constituting 5-10% of the marrow. Importantly, these biopsies revealed no abnormal clusters or increased numbers of blast cells. The image of the bone marrow biopsy can be seen in Figure 1.

Immunohistochemistry tests for CD20, CD3, and CD34 were negative, ruling out malignancies such as lymphoma and granuloma. Other standard investigations, including prostate-specific antigen (PSA), immunoglobulin A (IgA), immunoglobulin G (IgG), immunoglobulin M (IgM), erythrocyte sedimentation rate (ESR), hepatitis B surface antibody (anti-HBs), hepatitis C virus antibody (anti-HCV), antinuclear antibody (ANA), cytoplasmic antineutrophil cytoplasmic antibodies (c-ANCA), and perinuclear antineutrophil cytoplasmic antibodies (p-ANCA), returned normal results. Following these extensive but inconclusive findings, he received supportive care and corticosteroids before discharge. Initial laboratory tests, including a complete blood count, are detailed in Table 1.

**Table 1:** Laboratory test results of the patient.

Laboratory test	Value	Reference range
Hemoglobin	10.5 g/dL	14 – 18 g/dL
White blood cells (WBC)	4460/mm³	5000 – 10,000/mm³
Platelets	233,000/µL	150,000 – 450,000/µL
Mean corpuscular volume (MCV)	99.4 fL	80 – 100 fL
Reticulocyte count	1.5%	0.5 – 2.5%
Atypical blasts	Not detected	Not applicable
Prostate-specific antigen (PSA)	0.708 ng/mL	1.0 – 1.5 ng/mL
Immunoglobulin A (IgA)	3.1 g/L	0.8 – 4.00 g/L
Immunoglobulin G (IgG)	19 g/L	6.0 – 16.0 g/L
Immunoglobulin M (IgM)	1.9 g/L	0.5 – 2.00 g/L
Erythrocyte sedimentation rate (ESR)	120 mm/hr	<20 mm/hr
Hepatitis B surface antibody (anti-HBs)	Negative	Negative
Hepatitis C virus antibody (anti-HCV)	Negative	Negative
Antinuclear antibody (ANA)	Negative	Negative
Cytoplasmic antineutrophil cytoplasmic antibodies (c-ANCA)	Negative	Negative
Perinuclear antineutrophil cytoplasmic antibodies (p-ANCA)	Negative	Negative

Approximately three months after his initial discharge, the patient experienced recurring episodes of fever, unexplained weight loss, and a swollen left leg, accompanied by non-tender reddish nodules on both arms that appeared and disappeared spontaneously. These persistent and troubling symptoms necessitated another admission for further evaluation. Upon re-evaluation, his general physical examination highlighted pale skin and three to four red nodules on each arm, while the rest of the physical and systemic examinations showed no abnormalities.

Laboratory investigations revealed significant findings: an elevated ESR of 120 mm/hr, a low white blood cell count (WBC) of 3700 cells/µL, anemia with a hemoglobin (Hb) level of 9.21 g/dL, platelets at 321,000/µL, and a mean corpuscular volume (MCV) of 105 fL. Lactate dehydrogenase (LDH) was slightly elevated at 69 U/L. Additional diagnostic procedures, including a special smear and repeat bone marrow biopsy, yielded results consistent with previous findings, showing no new pathological indications.

Further diagnostic assessments were conducted to explore the range of symptoms exhibited by the patient. A Doppler ultrasound of the left leg revealed thrombosis in the femoral vein, though the remainder of the vasculature appeared normal. An esophagogastroduodenoscopy (EGD) identified candidiasis at the lower end of the esophagus, while a subsequent colonoscopy showed no abnormalities. Comprehensive viral screenings, including tests for hepatitis B surface antigen (anti-HBs), hepatitis C virus (anti-HCV), human immunodeficiency virus (HIV), and ANA, were all negative. Liver and kidney function tests, as well as serum electrolyte levels, were within normal limits.

Additionally, a lateral view X-ray of the skull did not reveal any lytic lesions, and tests for Bence-Jones proteins in the urine were negative, ruling out multiple myeloma. Serum protein electrophoresis confirmed the absence of abnormal proteins, and a CT scan of the chest, abdomen, and pelvis showed no noteworthy findings. Biopsies of the nodules were non-diagnostic, excluding erythema nodosum.

Given the lack of a definitive diagnosis and considering the prevalence of tuberculosis with atypical presentations in the region, a trial of anti-tuberculosis medication was initiated. However, after completing a six-month course of treatment, there was no significant improvement in his condition.

**Figure 2:**
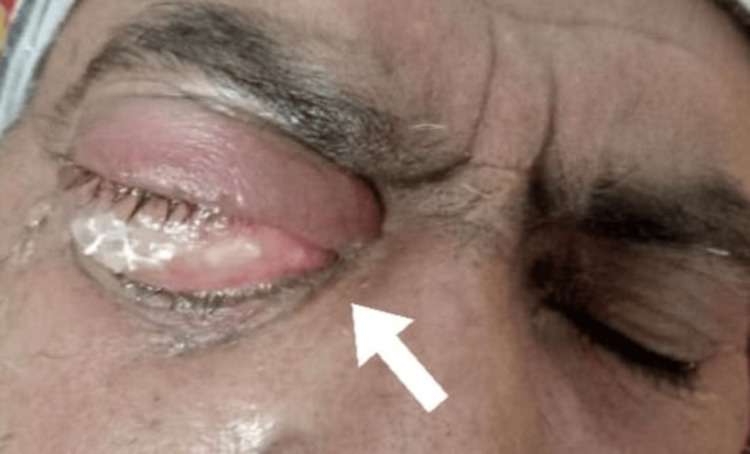
Lesion in the right eye before glucocorticoid therapy, as pointed by the arrow.

After completing a six-month regimen of anti-tuberculosis therapy, the patient was readmitted due to new symptoms: right orbital swelling and a persistent fever lasting 10 days. During examination, the affected eye was extremely tender to touch and exhibited severe ecchymosis. An MRI of the brain and orbits showed thickening in the right pre-septal region and mild fluid accumulation. Tests for p-ANCA and c-ANCA returned results within normal limits. An ophthalmologist was consulted, and treatment for suspected orbital cellulitis was initiated. Despite these measures, the patient was discharged without a definitive diagnosis of the root cause of his symptoms. The affected right eye is depicted in Figure 2.

**Figure 3:**
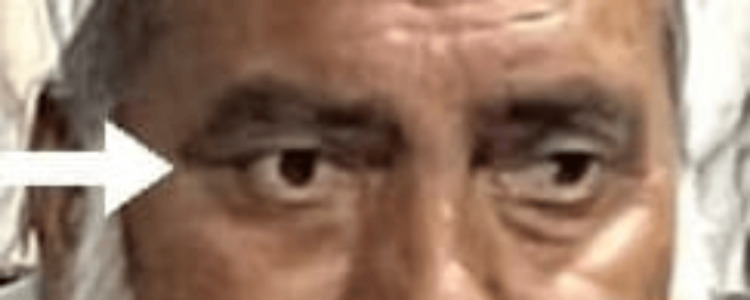
Right eye after administration of glucocorticoid therapy, as pointed by the arrow.

The patient soon returned to the healthcare facility with persisting issues: non-tender, recurrent nodules on his arm, and decreased vision. A thorough review of his medical records led to the consideration of a rare diagnosis, i.e., IgG4-RD. A serum human IgG4 level was measured, revealing a significantly elevated level of 1504 mg/L, well above the normal range of 39.2-864 mg/L. Based on these findings, he was diagnosed with IgG4-RD, and treatment was initiated with Solu-Medrol injections (250 mg) for four days, followed by oral prednisolone (0.6 mg/kg) for one month. This treatment initially led to improvement, but symptoms relapsed upon tapering the steroids. Consequently, rituximab therapy was started. The patient showed stable and improved health conditions at the six-month follow-up, as depicted in Figure 3.

The extended and complex diagnostic journey, spanning nearly two years to pinpoint this rare disease, highlights the significant challenges and intricacies inherent in diagnosing IgG4-RD. This case exemplifies the critical need for persistent investigative efforts and clinical vigilance to identify and manage such elusive medical conditions effectively.

## Discussion

The prevalence of IgG4-RD remains elusive due to diagnostic challenges, as the condition is frequently unrecognized or misdiagnosed. Epidemiological data from a cohort of 125 biopsy-confirmed cases show that the mean age at diagnosis is 55 years, with symptoms typically starting around 50 years of age; notably, 78% of these patients were White, and 61% were male, with head and neck tissues being the most commonly affected sites [7]. Additional data from the Mayo Clinic on 166 IgG4-RD patients revealed a median onset age of 61 years, with a male-to-female ratio of 3:1, and 80% being White; in this group, hepatobiliary manifestations were most frequent [8]. Contrastingly, a Japanese study of 114 cases reported a similar gender distribution but found an equal male-to-female ratio in IgG4-RD affecting head and neck areas (sialadenitis or dacryoadenitis), while the average age at diagnosis was higher, at 64 years [6]. Interestingly, IgG4-RD appears less prevalent among children, with a systematic review identifying only 22 cases, where the median age was 13 years (ranging from 22 months to 17 years), and a majority (64%) were female, indicating a shift from the adult disease’s male predominance. In pediatric cases, ophthalmic IgG4-RD emerged as the most common presentation, observed in 44% of the cases [9]. These data underscore the diverse demographic and clinical manifestations of IgG4-RD across different age groups and ethnicities, highlighting the complexities involved in its recognition and diagnosis.

Both human leukocyte antigen (HLA) and non-HLA genes are implicated in developing IgG4-RD, suggesting a genetic predisposition. An important aspect of its pathogenesis involves the dysfunction or reduction in regulatory T cells (Treg cells), which are critical in maintaining immune tolerance and preventing autoimmune disorders [10]. In IgG4-RD, these Treg cells play a crucial role, particularly through their influence on cytokine production. Elevated levels of IL-4, IL-5, and IL-13 are noted in patients and are associated with increased peripheral eosinophilia and IgE levels, which are common features of this disease. Furthermore, IL-10 is notably involved, enhancing IgG4 production and promoting fibrosis through the upregulation of transforming growth factor-beta (TGF-β), a key fibrogenic cytokine. Additionally, CD4+ cytotoxic T lymphocytes (CTLs) appear instrumental in the disease’s pathology, with an increase in plasmablasts that potentially reactivate these CTLs, prompting them to produce fibrosis-inducing cytokines like IL-1β and TGF-β1 [11]. This complex interplay of immune cells and cytokines underscores the intricate immune mechanisms driving IgG4-RD, contributing to its systemic manifestations and challenges in treatment.

The clinical manifestations of IgG4-RD are highly variable and depend significantly on the organs or tissues involved. The most frequent presentation involves mass lesions or noticeable organ enlargement. Organs commonly affected include the salivary and lacrimal glands, pancreas, biliary tract, and kidneys; however, IgG4-RD can potentially affect virtually any organ, and cases of multiorgan involvement are also documented [12]. The pathological process is characterized by inflammatory infiltration and progressive fibrosis, which not only impair organ function but also lead to tumor-like effects. These effects can manifest as obstructions or compression complications within the affected organs, contributing to the complexity and severity of the disease [13]. This broad spectrum of potential involvement necessitates a thorough and often extensive diagnostic approach to accurately identify and manage the disease across its various potential presentations.

Diagnosing IgG4-RD presents considerable challenges due to its nonspecific and variable clinical presentation, which can involve any organ system. The indistinct symptoms necessitate a comprehensive and methodical diagnostic approach to differentiate IgG4-RD from a range of other conditions that it can mimic. These conditions encompass a wide spectrum of diseases, including malignancies, lymphoproliferative disorders, antineutrophil cytoplasmic antibodies (ANCA)-associated vasculitis, sarcoidosis, Sjögren’s syndrome, and Castleman’s disease, among others [14]. Accurate diagnosis, therefore, involves identifying the hallmarks of IgG4-RD and rigorously excluding these other potential causes through a combination of clinical evaluation, laboratory testing, imaging, and histopathological analysis. This careful delineation is crucial to ensure that patients receive the most appropriate and effective treatment.

Biopsy remains the foundational diagnostic step for IgG4-RD. Histological features such as storiform fibrosis and obliterative phlebitis notably enhance the specificity of the diagnosis [13,14]. A conclusive diagnosis of IgG4-RD is established through a comprehensive correlation of clinical, laboratory, imaging, and histopathological findings without any malignancy or other disease processes that could account for the observed symptoms.

For patients displaying asymptomatic features of IgG4-RD, such as lymphadenopathy or minor submandibular gland enlargement, a monitoring approach without immediate treatment may be appropriate. However, most cases necessitate initiating treatment, especially when critical organs like the pancreas, biliary tree, aorta, mediastinum, kidneys, retroperitoneum, and mesentery are involved. In such scenarios, prompt intervention is crucial. Conditions like pachymeningitis, a serious manifestation of IgG4-RD, also demand urgent therapeutic measures [14].

Glucocorticoids are the primary treatment, expected to yield a rapid and favorable response. The initial dose of glucocorticoids is generally maintained for two to four weeks, followed by a gradual taper over three to six months, based on patient response. In cases where glucocorticoids cannot be tapered due to ongoing disease activity, the addition of a steroid-sparing agent such as methotrexate, azathioprine, mycophenolate, 6-mercaptopurine, or cyclophosphamide is considered. Rituximab (RTX) monotherapy has shown promise in inducing remission, significantly reducing both IgG4 levels and circulating plasmablast counts [15,16].

For maintenance therapy post-remission, particularly in patients at high risk of organ dysfunction or relapse, a regimen involving a steroid-sparing agent and rituximab is recommended. Although no consensus exists on the optimal duration or regimen for maintenance therapy, managing disease flares should include the reintroduction or escalation of glucocorticoids and, if necessary, initiating an immunosuppressive agent if one has not been used previously [14]. This comprehensive approach aims to stabilize the disease and prevent long-term complications.

We acknowledge that the inclusion of additional bone marrow and trephine biopsy images would further substantiate our findings. However, the image provided is the only one we could procure, and it effectively illustrates the key histopathological features observed in this case. Despite the limitation of having a single image, it adequately demonstrates the hypercellularity and absence of abnormal clusters or increased blast cells, thereby supporting our diagnostic conclusions. This constraint underscores the challenges in obtaining comprehensive visual documentation in clinical practice, yet we believe it does not compromise the integrity of our report.

## Conclusions

This case is a prime example of the difficult diagnostics associated with IgG4-RD, a rare and frequently perplexing illness. Even after much research and early misdiagnoses, getting higher serum IgG4 levels was essential to enabling focused therapy approaches. This instance demonstrates the need for a thorough assessment in handling difficult clinical presentations. It also highlights the need to add uncommon disorders to the differential diagnosis when faced with complicated patient symptoms, improving the chances of a correct diagnosis and successful therapy.
